# Dual fluorescent labelling of the human malaria parasite *Plasmodium falciparum* for the analysis of the ABC type transporter *pfmdr2*

**DOI:** 10.1186/1475-2875-11-371

**Published:** 2012-11-08

**Authors:** Benyamin Rosental, Uzi Hadad, Rosa Sinay, Alex Braiman, Angel Porgador, Yaakov Pollack

**Affiliations:** 1 The Shraga Segal Department of Microbiology and Immunology, Faculty of Health Sciences, Ben-Gurion University of the Negev, P.O. Box 653, Beer-Sheva, 84105, Israel; 2 National Institute for Biotechnology in the Negev, Ben-Gurion University of the Negev, Beer Sheva, Israel

**Keywords:** Fluorescent labelling, Malaria, Plasmodium falciparum, *pfmdr2*, Fluo-3/AM, Hoechst 33342, Heavy metals

## Abstract

**Background:**

The study of the *Plasmodium falciparum* heavy metal transporter gene *pfmdr2* employed radioactive labelled heavy metal. As the use of radioactive isotopes shrank considerably during the last few years, resulting in the cessation of the production of some isotopes, amongst them Cadmium^109^ which was used for that purpose, a different approach had to be developed. Herein, a dual fluorescent labelling of heavy metals accumulation in the *P. falciparum* parasite is proposed as an alternative to the use of radioactive labelled heavy metals.

**Methods:**

*Plasmodium falciparum* Cd resistant and sensitive strains at the trophozoite stage were used in this study. The cells were cultured at different CdCl_2_ concentrations and for different time periods followed by staining of the infected red blood cells with Fluo-3/AM for Cd detection and Hoechst 33342 for parasite DNA labelling. The fluorescent analysis was done by flow cytometry and confocal microscopy.

**Results:**

The results show that the sensitive strain has a higher Fluo-3/AM fluorescence in a Cd concentration and time dependent manner, whereas in the resistant strain Fluo-3/AM fluorescence levels were negligible and increased only at high concentrations of Cd and at long incubation periods, but to a much lesser extent than the sensitive strain**.** No Cd uptake is observed in uninfected red blood cells populations originating from cultures infected with either sensitive or resistant strain. In addition, confocal microscopy overlay of Fluo-3/AM and Hoechst staining shows that the Cd metal accumulates in the parasite itself.

**Conclusions:**

The dual fluorescent labelling is a valid method for detecting heavy metal accumulation in *P. falciparum*. Furthermore, in contrast to the use of radioactive labelled heavy metal, the fluorescent labelling enables us to differentiate between the different populations existing in a *P. falciparum* infected red blood cells cultures and thus actually study a phenomenon at the level of a single cell.

## Background

The development of the *in vitro* culturing technique of *Plasmodium falciparum* by Trager and Jensen [1] revolutionized the understanding of various aspects in the life cycle of this deadly parasite. The adoption of this technique in the research of malaria enabled the development of biochemical, physiological, immunological and genetic techniques [2-5] which resulted in a more comprehensive understanding of the biology of the parasite. In spite of this advancement, falciparum malaria remains the most prevalent infectious disease resulting in a high level of morbidity and mortality [6]. The major reasons for this state is the lack of an appropriate vaccine [7,8] and the evolving phenomenon of anti-malarial drug resistance [9,10]. The gaps which still exist in understanding the biology of the parasite contribute, although to a lesser extent, to this state. Since much of the studies on *P. falciparum* are done on the intact infected red blood cell (RBC) unit, it is preferable to be able to localize unequivocally the activity within the infected unit. This paper describes an assay of that nature which localizes heavy metal resistance to the parasite itself. By comparing heavy metal resistant and sensitive *P. falciparum* lines, it was previously shown [11] that this property, which prevents the accumulation of the heavy metal, is probably determined by the parasite's *P. falciparum* multidrug resistance 2 (*pfmdr2*) gene. However, as the labelling technique used a radioactive heavy metal, it was not possible to assess the resistance/sensitivity state at the level of a single infected RBC whereas the technique described in this paper allows it. This was achieved by dual labelling with Fluo-3/AM which labels the heavy metal whose transport is studied and Hoechst which labels the parasite by binding to its DNA. Fluo-3/AM was scarcely used in the study of *P. falciparum* and in those cases it served for determination of Ca^+2^ metabolism [12-15]. Hoechst and derivatives is used more commonly and in all cases for the visualization of the parasite e.g. [16-20]. No dual labelling with these two dyes was reported hitherto in the literature.

## Methods

### Parasites and culture conditions

The isolate *P. falciparum* FCR3 and a cadmium resistant line originating from it were used throughout this study. The *in vitro* culturing and synchronization of the parasites were carried out by standard protocols [1,21]. Briefly, the parasites were cultured in flasks at 37°C and 5% haematocrit in RPMI 1640 medium supplemented with human heat inactivated (30min at 56°C) plasma (A^+^ or AB^+^), 50μg/ml gentamycin, 25mM HEPES, and 0.25% sodium bicarbonate in an atmosphere of 5% O_2_ ,5% CO_2_ and 90% N_2_. The wild-type FCR3 line demonstrates sensitivity to heavy metal exposure, whereas the line originating from it by culturing it in the presence of a low concentration of CdCl_2_ exhibits a resistant phenotype, up to a concentration of 500 nM CdCl_2_[11] . This ability is not cadmium-dependent, and this line is therefore cultured in its absence [11]. The resistant line was then used in the comparative studies with the wild-type line unexposed to CdCl_2_.

### Cd uptake and staining procedure

*P. falciparum* Cd resistant and sensitive strains infected RBCs at the trophozoite stage were cultured in a U shaped 96-well plate (10^5^ cells/well) in 200μl for indicated times (5-45min, or for 20min for concentration dependent experiments) at 37°C with indicated CdCl_2_ concentrations (0.01-1μM, or 0.1μM for time dependent experiments), followed by three washes. Fluo-3/AM (Invitrogen detection technologies) staining was at a concentration of 5μM for 20min at 37°C, followed by two washes and incubation for 20min at 37°C. Hoechst 33342 (Fluka) staining was performed for 30min on ice at a concentration of 20μM. After staining, the cells were held on ice until analysed by flow cytometry and confocal microscopy. All procedures and washes were done in Dulbecco's Phosphate Buffered Saline (DPBS) (without Ca^2+^ and Mg^2+^) containing 3.5% glucose. The addition of glucose enabled the maintenance of the *pfmdr2* ATP dependent transporter activity. Experiments were done at 8-12% parasitaemia, where in each experiment parasitaemia for the sensitive and resistant strains were the same.

### Quantitation analysis

#### Flow cytometry

Stained infected RBCs were analysed by the fluorescence of Hoechst and Fluo-3/AM. Hoechst fluorescence was excited using a violet laser at 405nm and measured with a 450/50nm filter. Fluo-3/AM fluorescence was excited using a blue laser at 488nm and measured with a 530/30nm filter. Flow cytometry was performed using FACSCanto II (BD Bioscience) and results were analysed using FlowJo software (Tree Star).

#### Confocal microscopy

Stained infected RBCs were applied to a chambered μ-slide (ibiTreat, #80826, ibidi). The slides were mounted to the Olympus Fluoview FV1000 confocal microscope (Olympus: UPLSAPO 60X Oil NA:1.35). Fields containing infected RBCs were selected and images were acquired. Hoechst fluorescence was detected using a 405nm laser and measured in the 461nm channel; Fluo-3/AM fluorescence was detected using a 488nm laser and measured in the 527nm channel. Fields with infected RBCs were selected and images were acquired with indicated zoom magnitudes.

## Results and discussion

To evaluate the Cd uptake by the malaria parasite we used Cd labelled with the fluorescent dye Fluo-3/AM. Flow cytometry was performed for quantitative evaluation and confocal microscopy for qualitative measurement. Compared to the previous method of radioactive Cd detection [11], the fluorescent detection allows to differentiate between the fluorescence of the infected RBC and that of the non-infected one. This separation is of utmost importance as it allows the detection of low number of infected RBCs in the total cell population. For detection of infected RBCs we used the DNA dye Hoechst 33342. As RBCs lack DNA, the only population that will be stained will be that of the infected RBCs due to the staining of the parasite's DNA. Hoechst staining to identify RBC infected with malaria has been used in many studies e.g. [16–20].

**Figure 1:**
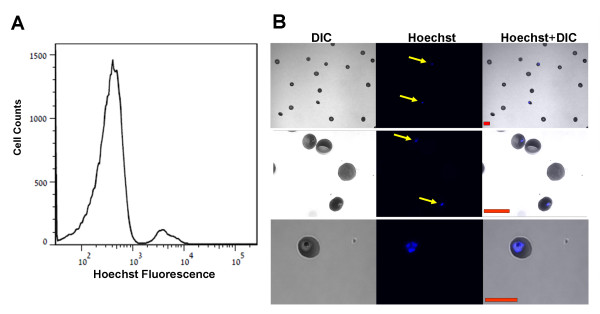
**Detection of *P. falciparum *infected RBCs using Hoechst staining.** (**A**) Representative flow cytometry fluorescent histogram of RBCs infected with *P. falciparum* stained with Hoechst. The Hoechst fluorescent axis is in Log scale. (**B**) RBCs infected with *P. falciparum* were stained with Hoechst, and then applied to chambered μ-slides. Representative confocal images are shown. Left panels: Differential Interference Contrast (DIC), middle panels: Hoechst (Blue), right panels: overly of Hoechst staining and DIC. Cells positive to Hoechst are marked with yellow arrows. [Red scale bar = 10μm].

Using this approach it can be seen that Hoechst staining clearly differentiates, by flow cytometry, between two RBCs populations of high and low Hoechst fluorescence (Figure 1A). Furthermore, analysis of these two populations using confocal microscopy shows unequivocally that: a) the high intensity Hoechst population consists of only infected RBCs, whereas the low intensity population contains only non-infected cells (Figure 1B, two upper panels). b) Only the parasite within the RBC is stained (Figure 1B, lower panels). Thus, the Hoechst staining enables us to focus directly on the infected RBCs.

**Figure 2:**
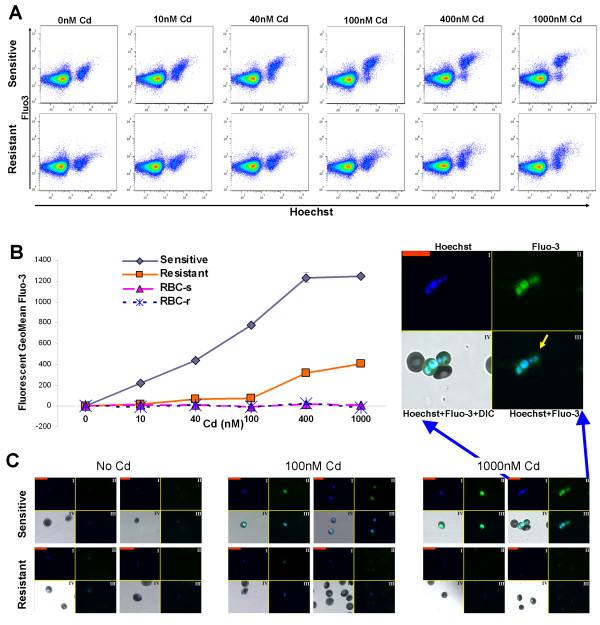
**Concentration dependent accumulation of Cd in RBCs infected with *P. falciparum *Cd resistant and sensitive strains.** (**A**-**B**) Fluo-3/AM fluorescence in Cd concentration dependent manner analysed by flow cytometry, Sensitive and Resistant denote RBCs infected with sensitive and resistant parasite respectively. (**A**) Two dimensional flow cytometry dot plots of stained populations of RBCs with Fluo-3/AM and Hoechst. (**B**) After gating the populations for positive and negative to Hoechst, at a threshold of 1.5*10^3^ Hoechst fluorescent intensity, Fluo-3/AM fluorescence geometric mean was calculated. The samples without Cd served as control. RBC-s and RBC-r denote non-infected RBCs originating from the sensitive and resistant cultures respectively. Each time point is a mean of duplicates, Bars ± SD. (**C**) Confocal images: I: Hoechst (Blue), II: Fluo-3/AM (Green), III: combined Hoechst and Fluo-3/AM, IV: combined Hoechst and Fluo-3/AM with DIC (also shown in the enlarged image). Yellow arrow points to Fluo-3/AM overlapping with Hoechst. [Red scale bar = 20μm]. For the sensitive and resistant strains parasitaemia was 11%. The results are from one representative experiment of three performed.

To assess the fluorescent method of Fluo-3/AM staining for Cd uptake, the *P. falciparum pfmdr2* system, an ABC-type transporter that is involved in heavy metal homeostasis [11] was used. In this system, the Cd-resistant strain has a functional form of *pfmdr2* gene, enabling its survival in the presence of high Cd concentrations. On the other hand, the sensitive strain has an impaired form of this gene, due to a premature translational termination, thus it cannot survive the high concentrations of Cd accumulated [11]. First, the uptake at different Cd concentrations was assessed. The results show that the sensitive strain has a higher Fluo-3/AM fluorescence in a Cd concentration dependent manner, while in the resistant strain Fluo-3/AM fluorescence levels increases only at high concentrations of Cd, but to a lesser extent than the sensitive line (Figure 2A-B). Similar results were obtained by confocal microscopy (Figure 2C). Furthermore, the confocal microscopy overlay of Fluo-3/AM and Hoechst staining (Figure 2C enlarged image) shows that the Fluo-3/AM and the Hoechst staining are localized within the parasite (yellow arrow). No Cd uptake is observed in uninfected RBCs populations originating from cultures infected either with the sensitive or the resistant lines (Figure 2A-C). These last two points demonstrate that the Cd uptake measured by Fluo-3/AM is specific to infected RBCs, and that this Cd uptake accumulates mainly within the parasite.

**Figure 3:**
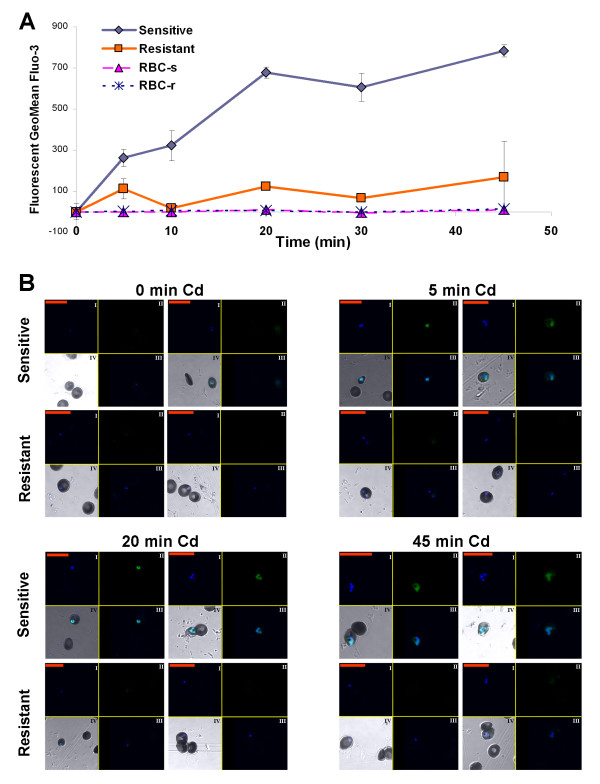
**Time dependent Cd accumulation in RBCs infected with *P. falciparum *Cd resistant and sensitive strains.** (**A**) Fluo-3/AM fluorescence in a time dependent manner analysed by flow cytometry. After gating the populations for positive and negative to Hoechst, Fluo-3/AM fluorescence geometric mean was calculated. The samples without Cd served as control. Sensitive and resistant denote RBCs infected with sensitive and resistant parasite respectively. RBC-s and RBC-r denote non-infected RBCs originating from the sensitive and resistant cultures respectively. Each time point is a mean of duplicates, Bars ± SD. (**B**) Confocal images: I: Hoechst (Blue), II: Fluo-3/AM (Green), III: combined Hoechst and Fluo-3/AM, IV: combined Hoechst and Fluo-3/AM with DIC. [Red scale bar = 20μm]. The results are from one representative experiment of two performed. For the sensitive and resistant strains parasitaemia was 10%.

To further characterize the Cd accumulation by Fluo-3/AM in comparison to the radioactive method, a time curve of Cd uptake experiments was performed. As measured by flow cytometry, Fluo-3/AM fluorescence increases in a time dependent manner in the RBCs infected with the *P. falciparum* sensitive line whereas the level of fluorescence in those infected with the resistant line was low and not time dependent (Figure 3A). In accordance with the results in Figure 2, the uninfected RBCs did not show any change in fluorescence levels with incubation time (Figure 3A). The confocal microscopy images confirmed the results obtained by flow cytometry (Figure 3B), namely, levels of Fluo-3/AM increase in the sensitive strain in a time dependent manner.

## Conclusions

The results demonstrate that Fluo-3/AM fluorescent labelling is a reliable method for following a physiological process occurring in living cells, in this case the active accumulation of Cd in *P. falciparum* infected RBCs. The method is time and concentration dependent, the two basic biological parameters needed for the characterization of a biological process. It is also shown that the use of Hoechst dye enables the distinction between the different populations existing in a *P. falciparum* infected RBCs culture and thus actually study a phenomenon at the level of a single cell. This is the major advantage of this procedure over the previous radioactive method which yielded information only on the entire population. The method described here can be used both for quantitative measurements (flow cytometry) as well as for qualitative assessments (confocal microscopy). As was shown recently for studying oxidative stress in *P. falciparum* infected RBCs [22], the basic idea underlying this method can be extended to the study of other properties of the parasite once the appropriate fluorescent tag is available.

## Abbreviations

RBCs: Red blood cells; DIC: Differential Interference Contrast; Cd: Cadmium/Cd^2+^; pfmdr2: *P. falciparum* multidrug resistance 2.

## Competing interests

The authors declare that they do not have any competing interests.

## Authors’ contributions

BR designed, performed and analysed the study, wrote the manuscript. UH and RS conducted experimental procedures in confocal analysis and culturing the parasites respectively. AB participated in confocal microscopy design and analysis of experiments. AP was involved in designing experiments and critical evaluation of the manuscript. YP conceptualized the study, designed the experiments, analysed the results and wrote the manuscript. All authors read and approved the final manuscript.

## References

[B1] Trager W Jensen JB Human malaria parasites in continuous culture Science 1976 193 673 675 10.1126/science.781840 781840

[B2] Ljungström I Perlmann H Schlichtherle M Scherf A Wahlgren M Methods in malaria research 2004 4 http://www.mr4.org/Portals/3/Pdfs/ProtocolBook/Methods_in_malaria_research.pdf.

[B3] Grimberg BT Methodology and application of flow cytometry for investigation of human malaria parasites J Immunol Meth 2011 367 1 16 10.1016/j.jim.2011.01.015 PMC3071436 21296083

[B4] Campuzano-Zuluaga G Hänscheid T Grobusch MP Automated haematology analysis to diagnose malaria Malar J 2010 9 346 10.1186/1475-2875-9-346 21118557 PMC3013084

[B5] Crabb BS Rug M Gilberger T-W Thompson JK Triglia T Maier AG Cowman AF Transfection of the human malaria parasite *Plasmodium falciparum* Meth Mol Biol 2004 270 263 276 10.1385/1-59259-793-9:263 15153633

[B6] Snow R Guerra C Noor A Myint H Hay S The global distribution of clinical episodes of *Plasmodium falciparum* malaria Nature 2005 10 214 217 15759000 10.1038/nature03342 PMC3128492

[B7] Schwartz L Brown GV Genton B Moorthy VS A review of malaria vaccine clinical projects based on the WHO rainbow table Malar J 2012 11 11 10.1186/1475-2875-11-11 22230255 PMC3286401

[B8] Thera MA Plowe CV Vaccines for malaria: how close are we? Ann Rev Med 2012 63 345 357 10.1146/annurev-med-022411-192402 22077719 PMC3338248

[B9] White NJ Antimalarial drug resistance J Clin Invest 2004 113 1084 1092 15085184 10.1172/JCI21682 PMC385418

[B10] Sá JM Chong JL Wellems TE Malaria drug resistance: new observations and developments Essays Biochem 2011 51 137 160 22023447 10.1042/bse0510137 PMC3428136

[B11] Rosenberg E Litus I Schwarzfuchs N Sinay R Schlesinger P Golenser J Baumeister S Lingelbach K Pollack Y pfmdr2 confers heavy metal resistance to *Plasmodium falciparum* J Biol Chem 2006 281 27039 27045 10.1074/jbc.M601686200 16849328

[B12] Varotti FP Beraldo FH Gazarini ML Garcia CRS *Plasmodium falciparum* malaria parasites display a THG-sensitive Ca2+ pool Cell Calcium 2003 33 137 144 10.1016/S0143-4160(02)00224-5 12531190

[B13] Gazarini ML Garcia CRS The malaria parasite mitochondrion senses cytosolic Ca2+ fluctuations Biochem Biophys Res Commun 2004 321 138 144 10.1016/j.bbrc.2004.06.141 15358226

[B14] Niemoeller OM Foller M Lang C Huber SM Lang F Retinoic acid induced suicidal erythrocyte death Cellular Physiol Biochem 2008 21 193 202 10.1159/000113761 18209486

[B15] Bagnaresi P Barros NM Assis DM Melo PM Fonseca RG Juliano MA Pesquero JB Juliano L Rosenthal PJ Carmona AK Gazarini ML Intracellular proteolysis of kininogen by malaria parasites promotes release of active kinins Malar J 2012 11 156 10.1186/1475-2875-11-156 22564457 PMC3407703

[B16] Howard RJ Battye FL Mitchell GF Plasmodium-infected blood cells analyzed and sorted by flow fluorimetry with the deoxyribonucleic acid binding dye 33258 Hoechst J Histochem Cytochem 1979 27 803 813 10.1177/27.4.87413 87413

[B17] Reinders PP van Vianen PH van der Keur M van Engen A Janse CJ Tanke HJ Computer software for testing drug susceptibility of malaria parasites Cytometry 1995 19 273 281 10.1002/cyto.990190312 7537650

[B18] Grimberg BT Erickson JJ Sramkoski RM Jacobberger JW Zimmerman PA Monitoring *Plasmodium falciparum* growth and development by UV flow cytometry using an optimized Hoechst-thiazole orange staining strategy Cytometry A 2008 73 546 554 18302186 10.1002/cyto.a.20541 PMC3619977

[B19] Theron M Hesketh RL Subramanian S Rayner JC An adaptable two-color flow cytometric assay to quantitate the invasion of erythrocytes by *Plasmodium falciparum* parasites Cytometry A 2010 77 1067 1074 20872885 10.1002/cyto.a.20972 PMC3047707

[B20] Malleret B Claser C Ong ASM Suwanarusk R Sriprawat K Howland SW Russell B Nosten F Rénia L A rapid and robust tri-color flow cytometry assay for monitoring malaria parasite development Sci Rep 2011 1 118 22355635 10.1038/srep00118 PMC3216599

[B21] Lambros C Vanderberg JP Synchronization of *Plasmodium falciparum* erythrocytic stages in culture J Parasitol 1979 65 418 420 10.2307/3280287 383936

[B22] Fu Y Tilley L Kenny S Klonis N Dual labeling with a far red probe permits analysis of growth and oxidative stress in *P. falciparum*-infected erythrocytes Cytometry A 2010 77 253 263 20091670 10.1002/cyto.a.20856

